# Urchin-like Ce(HCOO)_3_ Synthesized by a Microwave-Assisted Method and Its Application in an Asymmetric Supercapacitor

**DOI:** 10.3390/molecules29020420

**Published:** 2024-01-15

**Authors:** Qing He, Wanglong Wang, Guohua Li, Wenmiao Chen, Xing Yang, Chengyuan Ni, Xing Fang

**Affiliations:** 1 Key Laboratory of Air-Driven Equipment Technology of Zhejiang Province, Quzhou University, Quzhou 324000, China; yang7092481@163.com (X.Y.); nichengyuan1@126.com (C.N.); 2 Department of Mechanical Engineering, Zhejiang University of Technology, Hangzhou 310058, China; 221122020226@zjut.edu.cn (W.W.); 15714178548@163.com (W.C.); 3 R&D Department, Quzhou Hixee Electronic Technology Co., Ltd., Quzhou 324000, China; bullcar@163.com

**Keywords:** Ce(HCOO)_3_, microwave-assisted, irradiation time, asymmetric supercapacitor

## Abstract

In this work, a series of urchin-like Ce(HCOO)_3_ nanoclusters were synthesized via a facile and scalable microwave-assisted method by varying the irradiation time, and the structure–property relationship was investigated. The optimization of the reaction time was performed based on structural characterizations and electrochemical performances, and the Ce(HCOO)_3_-210 s sample shows a specific capacitance as high as 132 F g^−1^ at a current density of 1 A g^−1^. This is due to the optimal mesoporous hierarchical structure and crystallinity that are beneficial to its conductivity, offering abundant Ce^3+^/Ce^4+^ active sites and facilitating the transportation of electrolyte ions. Moreover, an asymmetric supercapacitor based on Ce(HCOO)_3_//AC was fabricated, which delivers a maximum energy density of 14.78 Wh kg^−1^ and a considerably high power density of 15,168 W kg^−1^. After 10,000 continuous charge–discharge cycles at 3 A g^−1^, the ASC device retains 81.3% of its initial specific capacitance. The excellent comprehensive electrochemical performance of this urchin-like Ce(HCOO)_3_ offers significant promise for practical supercapacitor applications.

## 1. Introduction

Nanostructured materials have attracted great attention in recent decades due to their active domains on the surface compared to their corresponding bulk materials [1,2,3]. The hierarchical self-assembly of nanocrystals typically enhances mass transport, reduces diffusion resistance, and increases the surface area for adsorption and electrochemical reactions. Thus, it is particularly suitable for application in energy storage systems [4,5,6,7]. Batteries and supercapacitors are the most widely used energy storage devices. Supercapacitors have many notable advantages, including a much higher power density than rechargeable batteries and a superior energy density to conventional capacitors [8,9,10]. Electrodes are key components of supercapacitors, and their design and synthesis play a dominant role in factors affecting the performance of the device. Traditional electrode materials include carbon-based materials, conductive polymers, and transition metal oxides, which have their own advantages and disadvantages [11,12,13]. Although many researchers have made significant contributions to improving the performance of supercapacitor electrodes, there are still some common constraints. Commercial supercapacitors are mainly based on carbon-based electrodes, which have a low energy density due to the inherent characteristics of their electric double-layer capacitive (EDLC) mechanism [14,15,16]. Conductive polymers are prone to swelling and disintegrating during charge–discharge reactions [17,18,19]. As to electrode materials based on redox reactions, i.e., mainly nickel-/cobalt-based oxides, hydroxides, and sulfides, they usually have poor cycling stability [20,21,22,23,24]. Metal–organic compounds are a new type of attractive electrode materials by virtue of their rich metal redox sites, diverse topological structures, adjustable aperture, and optimizable synthesis parameters [25,26,27]. These materials have the potential to achieve a high energy density coupled with an excellent power density and long cycling life.

There are many options for the metal sources of metal–organic compounds. The most prominent sources are transition metal elements such as Ni, Co, Mn, Fe, Cu, Cr, V, Ce, etc. Among them, Ce–organic compounds are research hotspots in the fields of catalysis and electrochemical energy storage due to their extraordinary redox capability [28,29,30]. Benefitting from the unique electronic structure of rare earth metals and the 4f orbitals, the Ce^3+^ and Ce^4+^ can undergo rapid and reversible transitions in Ce–organic compounds, resulting in efficient electron conduction and high theoretical capacitance. For instance, Singh et al. synthetized three lanthanide-based metal−organic frameworks (MOFs) by the hydrothermal method, employing Ce, Sm, and Eu as the central metal ions and 5-nitroisophthalic acid (H_2_L) as the ligand. All three nanoporous MOFs had similar 3D crystal structures with distorted monocapped square antiprism geometries, and the Ce-H_2_L delivered the highest specific capacitance and energy storage capability in the fabricated symmetric supercapacitor (SSC) [31]. Kumaresan et al. obtained a rough spherical Ce-MOF using terephthalic acid (TPA) as the organic linker. The Ce-MOF was endorsed for SSC and achieved an energy density of 0.56 Wh kg^−1^ and power density of 10.6 kW kg^−1^ at 2 A g^−1^ [32]. In addition, Yoon et al. prepared a novel 1D cerium hydrogen phosphate (Ce(HPO_4_)_2_·xH_2_O) with a nanorod-like structure along with particles and clusters. The SSC based on Ce(HPO_4_)_2_·xH_2_O//Ce(HPO_4_)_2_·xH_2_O showed a specific energy of 2.08 Wh kg^−1^, specific power of 499.88 W kg^−1^, and 92.7% capacitance retention after 5000 cycles [33]. Overall, these studies demonstrate Ce–organic compounds with various nanostructures and their significant potential for application in the electrochemical field.

However, all the above-mentioned Ce–organic compounds were synthesized by hydro/solvothermal methods, and the reaction time ranges from 12 to 72 h. This poor efficiency has long been a concern for researchers. Recently, the microwave-assisted technique has been proven to be an effective method for rapidly synthesizing nanostructured materials [34,35,36]. Different from the slow heat transfer in traditional hydro/solvothermal methods, microwave-assisted synthesis utilizes electromagnetic waves to directly interact with polar molecules in the solution, with a dielectric mode to generate heat. Except for the instantaneous and rapid heating that is achieved, it also holds many other advantages such as a high bulk reaction temperature homogeneity, selective heating, and preparing nanomaterials in quantitative yields with high precision. Cho et al. successfully synthesized a high-quality Co-MOF-74 crystal in 1 h by microwave heating instead of 24 h via the solvothermal method. The crystal structure characteristics of these two samples are almost the same, but the average particle size of the former is much smaller than that of the latter [37,38]. Luo et al. prepared uniform Ni_3_(HCOO)_6_ particles with high crystallinity via the simple and fast microwave-assisted method for supercapacitor electrodes. Compared with the pristine Ni_3_(HCOO)_6_ ellipsoidal particles obtained by the solvothermal procedure, the reaction time was shortened from 48h to 30min, and the Ni_3_(HCOO)_6_ from the microwave-assisted method exhibited higher electrochemical reactivity [39,40]. However, there are very limited studies on the microwave-assisted synthesis of Ce–organic compounds and the provision of a scientific rationale between synthesis conditions, product phase structure, morphology, and the final electrochemical performance for supercapacitors that are based on them.

Herein, an urchin-like Ce(HCOO)_3_ electrode material was efficiently synthesized by the microwave-assisted method, with the formic acid (HCOOH) serving as the ligand. The process conditions were optimized by synthesizing Ce(HCOO)_3_ clusters at 300 W for different irradiation times of 150, 210, and 270 s, respectively. The obtained Ce(HCOO)_3_ clusters were characterized for structure, morphology, and electrochemical performances, and a correlation analysis was conducted of the structure–property relationship. Considering that an asymmetric supercapacitor (ASC) can fully utilize the potential windows of both the pseudocapacitive (PC) electrode and the EDLC electrode to maximize the energy density, the urchin-like Ce(HCOO)_3_, derived under the optimum irradiation time, was employed to fabricate Ce(HCOO)_3_//AC-based ASC. The cyclic voltammetry (CV), galvanostatic charge–discharge (GCD), electrochemical impedance spectroscopy (EIS), and cycling stability of the device were investigated. The energy density and power density of the ASC hold a significant advantage over many recently reported MOF-based devices. This work will contribute to the simple and effective synthesis of Ce(HCOO)_3_ nanostructures and promote the prospects for the electrochemical applications of the Ce–organic compounds.

## 2. Results and Discussion

**Figure 1:**
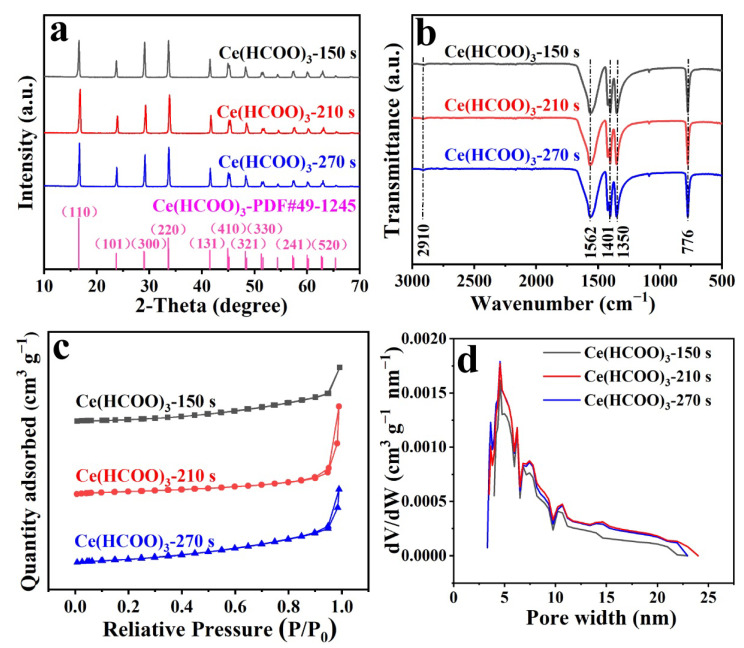
(**a**) XRD patterns, (**b**) FT-IR spectra, (**c**) N_2_ adsorption–desorption isotherms, and (**d**) the corresponding BJH pore size distribution curves of the Ce(HCOO)_3_-150 s, Ce(HCOO)_3_-210 s, and Ce(HCOO)_3_-270 s samples.

A series of Ce(HCOO)_3_ electrode materials were synthesized by a facile microwave-assisted method at an irradiation power of 300 W for different reaction times. Correspondingly, the samples were named Ce(HCOO)_3_-x (x represents the reaction time), i.e., Ce(HCOO)_3_-150 s, Ce(HCOO)_3_-210 s, and Ce(HCOO)_3_-270 s, respectively. Their X-ray diffraction (XRD) patterns are shown in Figure 1a. It can be observed that all the samples show distinct characteristic peaks at the same positions of 2θ = 16.45°, 23.56°, 28.91°, 33.51°, 41.33°, 44.76°, 48.10°, 51.16°, 57.20°, and 62.78°, corresponding to the (110), (101), (300), (220), (131), (410), (321), (330), (241), and (520) lattice planes of the Ce(HCOO)_3_ (JCPDS card No. 49-1245), respectively. The XRD patterns have no other miscellaneous peaks and comply exactly with the crystal database, which is a valid proof that the high-purity rhombohedral phase of Ce(HCOO)_3_ can be obtained easily via the microwave-assisted method. However, the intensity and sharpness of the XRD peaks for samples that were obtained at different reaction times vary, indicating that their crystallinity and grain sizes are different. The crystallinity indexes (CIs) of the samples were calculated by Person’s method [41] using Formula (1) as follows:

(1) $$CI=\frac{a+b+c}{l}=\frac{h\left(410\right)+h\left(401\right)+h(012)}{h(131)}$$

where the definitions of a, b, c, and l are depicted in Figure S1 (Supplementary Information). a, b, and c represent the heights of (410), (401), and (012) peaks between the crest and trough, while l represents the height of the main (131) peak from the crest to the baseline, set between 41° and 46°.

The crystallite sizes of the samples were determined using the classic Scherrer formula:

(2) $$D=\frac{0.89\;\lambda }{\beta \;cos\theta }$$

where D, λ, β, and θ represent the average size of the grain perpendicular to the lattice plane direction (Å), the X-ray wavelength (for Cu Kα radiation here, it is 1.5406 Å), the full width at half maxima (FWHM) of the selected diffraction peak (rad), and the Bragg diffraction angle in degree, respectively.

The CI of the Ce(HCOO)_3_ samples is found to ascend first and then decline with the increase in reaction time, as the calculation results of the Ce(HCOO)_3_-150 s, Ce(HCOO)_3_-210 s, and Ce(HCOO)_3_-270 s are 39.24%, 86.67%, and 65.93%, respectively (as given in Table 1). Meanwhile, the grain sizes of the Ce(HCOO)_3_-150 s, Ce(HCOO)_3_-210 s, and Ce(HCOO)_3_-270 s samples are calculated to be 34.27 nm, 44.19 nm, and 40.85 nm, which is line with the variation in CI as a function of the reaction time. The increase in grain size in the early stage is because the grain is in the period of growing, and the decrease in grain size after the reaction time exceeds 210 s is believed to be related to Ostwald ripening [42,43]. Based on these results, the Ce(HCOO)_3_-210 s has the highest crystallinity within a larger grain range, which is beneficial for its node-to-node charge-hopping behavior and achieving high conductivity, thereby enhancing its electrochemical performance [44].

Figure 1b displays the Fourier transform infrared spectroscopy (FT-IR) spectra of the Ce(HCOO)_3_-150 s, Ce(HCOO)_3_-210 s, and Ce(HCOO)_3_-270 s samples, respectively. A series of characteristic absorption bands centered at ~2910 cm^−1^ and ~1350 cm^−1^ can be related to the C−H stretching and bending vibrations. The characteristic absorption bands at ~1562 cm^−1^ and ~1401 cm^−1^ can be ascribed to the asymmetric and symmetric stretching mode of COO−. And the sharp absorption band at ~776 cm^−1^ belongs to the stretching vibrations of the Ce−O bond. The samples that have been irradiated for different times exhibit similar FT-IR spectra despite some slight peak shifts, confirming the formation of the same Ce(HCOO)_3_ substances [45]. This is in good agreement with the results of the XRD analysis.

To investigate the porosity of the Ce(HCOO)_3_ hierarchical structures that were synthesized for different reaction times, the N_2_ adsorption/desorption experiment at 77 K was performed on the Ce(HCOO)_3_-150 s, Ce(HCOO)_3_-210 s, and Ce(HCOO)_3_-270 s samples. The obtained isotherms are shown in Figure 1c. All three samples demonstrate characteristic adsorption/desorption isotherms of the Langmuir type IV according to the IUPAC classification. The typical H3-type hysteresis loop in the relatively high pressure (P/P_0_) region confirms the mesoporous structure [46]. The specific surface areas (SSAs) of the Ce(HCOO)_3_-150 s, Ce(HCOO)_3_-210 s, and Ce(HCOO)_3_-270 s samples, calculated by the Braunauer–Emmett–Teller (BET) method, are 1.93, 2.89, and 2.57 m^2^ g^−1^, respectively (as given in Table 2). These results verify that the irradiation time of the microwave-induced Ce(HCOO)_3_ samples can tune their surface and texture properties, which is also related to their crystal growth process. The highest SSA indicates that the Ce(HCOO)_3_-210 s sample exposes the most active sites to contact with electrolyte ions and participates in redox reactions, which may lead to the highest specific capacitance. In addition, the corresponding pore size distribution curves of all three samples by the Barrett–Joyner–Halenda (BJH) method are demonstrated in Figure 1d. It can be seen that Ce(HCOO)_3_ samples that have been synthesized for different irradiation time have similar pore size distribution ranges from 2.8 to 25 nm. The accumulated pore volumes of the Ce(HCOO)_3_-150 s, Ce(HCOO)_3_-210 s, and Ce(HCOO)_3_-270 s samples are 0.015, 0.023, and 0.020 m^3^ g^−1^, and the average pore sizes are 3.796, 3.391, and 3.803 nm, respectively. The largest pore volume and smallest mesopores of a Ce(HCOO)_3_-210 s sample would facilitate electrolyte penetration and provide enhanced participation of all the active sites inside the material; thus, the Ce(HCOO)_3_-210 s is theoretically expected to promise the best electrochemical performance among the different samples.

**Figure 2:**
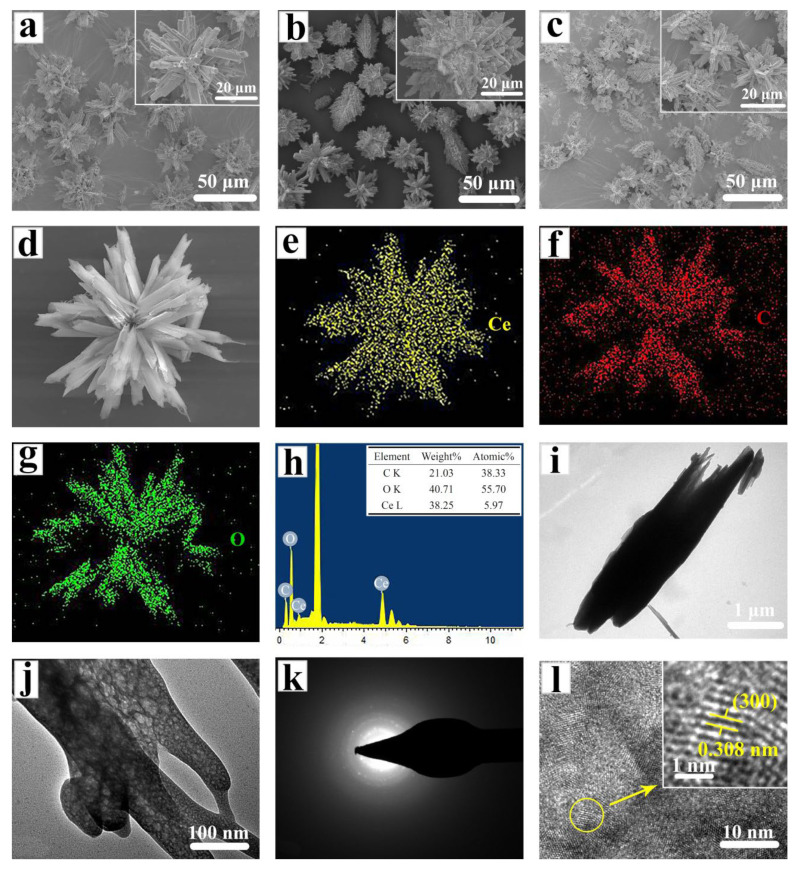
SEM images of the urchin-like (**a**) Ce(HCOO)_3_-150 s; (**b**) Ce(HCOO)_3_-210 s; and (**c**) Ce(HCOO)_3_-270 s samples. (**d**) EDS images of the Ce(HCOO)_3_-210 s with elemental mappings for (**e**) Ce; (**f**) C; (**g**) O; and (**h**) EDS analysis of Ce(HCOO)_3_-210 s. (**i**,**j**) TEM images of the sub-unit of urchin-like Ce(HCOO)_3_-210 s at different magnifications and (**k**) the corresponding SAED patterns. (**l**) HRTEM image of Ce(HCOO)_3_-210 s with the inset interplanar spacing of the lattice fringes.

The morphologies of the Ce(HCOO)_3_-150 s, Ce(HCOO)_3_-210 s, and Ce(HCOO)_3_-270 s samples were identified with scanning electron microscopy (SEM), and the resultant images are presented in Figure 2a–c. All the three images show the self-assembly of Ce(HCOO)_3_ rods to form 3D urchin-like clusters, signifying their similar morphological behaviors. The size of the Ce(HCOO)_3_ clusters varies from 20 to 50 μm and is dependent on the reaction time. In addition to the main urchin-like Ce(HCOO)_3_ clusters, there are also some scattered Ce(HCOO)_3_ particles in the SEM images. Compared to the precise 3D hierarchical structure of the urchin-like Ce(HCOO)_3_ clusters, which can facilitate electrolyte penetration, the scattered Ce(HCOO)_3_ particles are prone to being trapped in “dead volume” derived from isolated pores and unable to make contact with electrolyte ions, thereby hindering the electrochemical performance of supercapacitors [47]. It can be clearly seen from the magnified part (inset in each SEM images) that the essential difference of the three samples lies in the size of the sub-units, i.e., the Ce(HCOO)_3_ rods. The rods that constitute Ce(HCOO)_3_-150 s are slightly larger than those of Ce(HCOO)_3_-270 s and much larger than those of Ce(HCOO)_3_-210 s, resulting in the most comprehensive fine structure being that of Ce(HCOO)_3_-210 s. The smaller the size of the Ce(HCOO)_3_ sub-units is, the more abundant Ce^3+^/Ce^4+^ active sites can it provide at the same mass. These results match well with the BET analysis. In addition, the higher the fineness of the urchin-like hierarchical structure of the sample is, the more conducive it is to the migration of the electrolyte ions, which would reduce the interfacial charge transfer resistance (R_ct_) and thus enhance its electrochemical performance. Therefore, the potential of the Ce(HCOO)_3_-210 s for being the best electrode material among these samples is also supported by the morphological analysis. Accordingly, the Ce(HCOO)_3_-210 s sample was selected for the energy-dispersive X-ray spectroscopy (EDS) mapping analysis, and the results are shown in Figure 2d–h. The elemental mapping images reveal that Ce (yellow), C (red), and O (green) are homogeneously distributed in the urchin-like clusters. And the presence of Ce, C, and O elements can also be verified by the EDS spectrum. Further, the sub-unit of the urchin-like Ce(HCOO)_3_-210 s structure was investigated using transmission electron microscopy (TEM), and the images are displayed in Figure 2i–l. The bright-field TEM image in Figure 2i exhibits the debris of a fractured Ce(HCOO)_3_ rod, which can be seen from the fracture site as a bundle composed of smaller Ce(HCOO)_3_ nanorods. The magnified TEM image in Figure 2j assigns the Ce(HCOO)_3_ nanocrystals a size of 30–50 nm, which are in good agreement with the results calculated by the Scherrer formula in the XRD analysis. The corresponding selected area electron diffraction (SAED) pattern shown in Figure 2k reveals the polycrystalline nature of Ce(HCOO)_3_-210 s. Moreover, the high-resolution transmission electron microscopy (HRTEM) was employed to determine the interplanar spacing of the lattice fringes of the Ce(HCOO)_3_-210 s. As shown in Figure 2l, the well-defined atomic arrangement is indicative of the highly crystalline nature of the sample, which is in line with the calculated CI results. From the magnified inset part, a lattice spacing of 0.308 nm can be observed, and this matches well with the (3 0 0) crystal plane separation of the Ce(HCOO)_3_ crystal, confirming the composition of the microwave-induced products.

**Figure 3:**
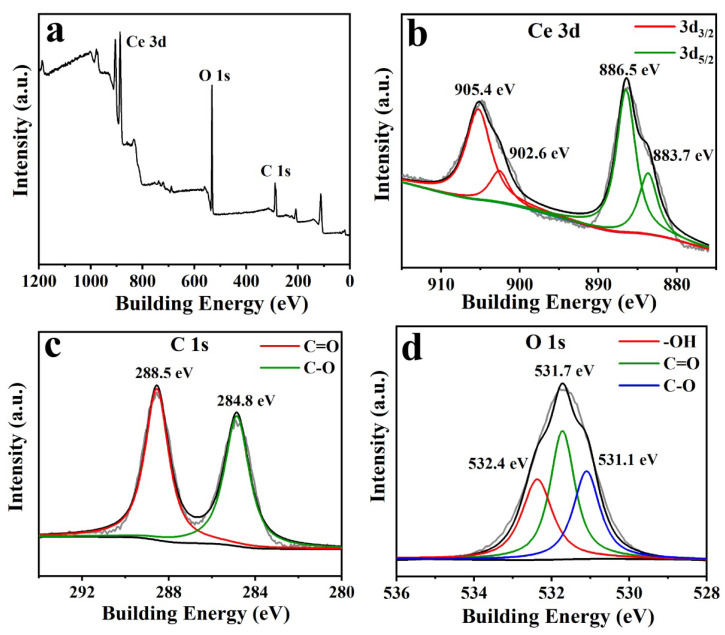
(**a**) X-ray photoelectron spectra (XPS) survey spectrum of the as-synthesized Ce(HCOO)_3_-210 s and high-resolution XPS scan of (**b**) Ce 3d, (**c**) C 1s, and (**d**) O 1s binding energies.

In order to further support the above structural characterizations and analyses, X-ray photoelectron spectroscopy (XPS) is performed on the Ce(HCOO)_3_-210 s sample to determine its surfaces’ chemical compositions and corresponding valence states. As shown in Figure 3a, the XPS survey spectrum confirms that the sample is mainly composed of Ce, O, and C elements. Moreover, the high-resolution XPS spectra of Ce 3d, C 1s, and O 1s are presented in Figure 3b–d. It can be seen from the Ce 3d spectrum that there are two main characteristic peaks at 905.4 eV and 886.5 eV, accompanied by satellite peaks at 902.6 eV and 883.7 eV, which correspond to the binding energy of Ce 3d_3/2_ and Ce 3d_5/2_, respectively. These results determine that cerium exists in the form of Ce^3+^. The characteristic C 1s signals at 288.5 eV and 284.8 eV belong to C=O and C−O, respectively. The O 1s spectrum can be deconvoluted into three peaks located at 532.4 eV, 531.7 eV, and 531.1 eV, which can be related to the various oxygen environments for −OH, C=O, and C−O, respectively. These observations clearly illustrate the electronic spin states of different elements in the Ce(HCOO)_3_ sample and align well with earlier research reports [48].

**Figure 4:**
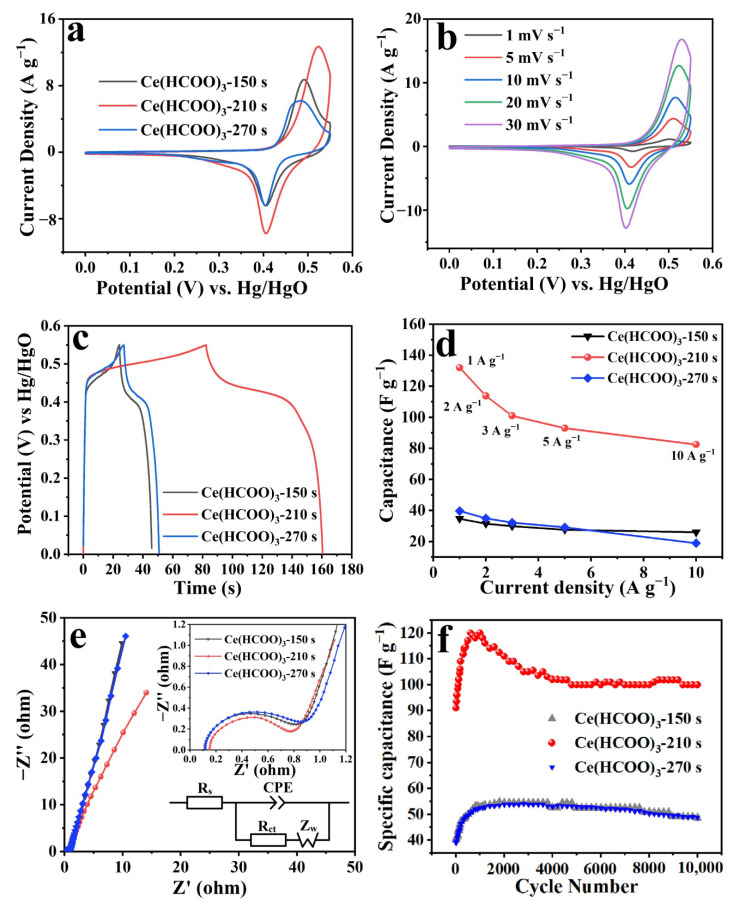
(**a**) Comparative CV curves of Ce(HCOO)_3_-150 s, Ce(HCOO)_3_-210 s, and Ce(HCOO)_3_-270 s at a scan rate of 20 mV s^−1^. (**b**) CV curves of Ce(HCOO)_3_-210 s at different scan rates. (**c**) Comparative GCD curves of all three samples at current density of 1 A g^−1^. (**d**) The specific capacitance of all three electrodes as a function of current density. (**e**) Comparative Nyquist impedance spectra and (**f**) cyclic performances at 10 A g^−1^ for 10,000 charge–discharge cycles of all three samples.

The electrochemical charge storage properties of the Ce(HCOO)_3_-150 s, Ce(HCOO)_3_-210 s, and Ce(HCOO)_3_-270 s electrodes are detected by the classic three-electrode system using Hg/HgO as reference electrode and Pt foil as counter electrode in a 3 M KOH aqueous electrolyte. The cyclic voltammetry (CV) investigations were performed at different scan rates within a potential window of 0–0.55 V. The comparative CV curves of Ce(HCOO)_3_-150 s, Ce(HCOO)_3_-210 s, and Ce(HCOO)_3_-270 s electrodes at 20 mV s^−1^ and the individual CV curves of Ce(HCOO)_3_-210 s at scan rates varying from 1 to 50 mV s^−1^ are displayed in Figure 4a,b. All the electrode materials demonstrate well-defined pairs of redox peaks, which originate from the typical faradaic behavior. The anodic peaks are located at 0.48–0.52 V, which can be attributed to the oxidation of Ce^3+^ to regenerate Ce^4+^ sites. The cathodic peaks centered around 0.40 V are due to the reduction of active Ce^4+^ to form Ce^3+^ sites. This reversible transition between different oxidation states of Ce^3+^/Ce^4+^ in Ce(HCOO)_3_ can be described as in Equation (3), given below:

(3) $${Ce}^{Ⅲ}+{OH}^{-}⇋{Ce}^{Ⅳ}(OH{)}_{ad}+e^{-1}$$

The area under the CV curves denotes the capacity that is obtained from the contribution of active electrode materials. It can be clearly observed from Figure 4a that the area under the CV curve of Ce(HCOO)_3_-210 s is much larger than those of Ce(HCOO)_3_-270 s and Ce(HCOO)_3_-150 s. In Figure 4b, the contour of the CV curves remains almost the same with an increase in the scanning rate, signifying the ability to charge–discharge at a faster rate and excellent kinetic reversibility of the Ce(HCOO)_3_-210 s electrode. The slight shifts of the anodic and cathodic peaks to higher and lower potentials are caused by the Ohmic resistance and the polarization effect in nature [49].

Then, the galvanostatic charge–discharge (GCD) tests were performed to directly examine the specific capacitances of the Ce(HCOO)_3_-150 s, Ce(HCOO)_3_-210 s, and Ce(HCOO)_3_-270 s samples. The comparative GCD curves of all three materials at 1 A g^−1^ are exhibited in Figure 4c, and the GCD curves for them at various current densities are shown in Figure S2. The quasi-triangle symmetric geometry and poorly defined plateaus of the GCD profiles are the characteristics of excellent reversible faradaic processes. Notably, it can be seen from Figure 4c that Ce(COOH)_3_-210 s demonstrates a much longer discharging time, followed by Ce(COOH)_3_-270 s and then Ce(COOH)_3_-150 s, which is in line with the CV profiles and indicates a better charge-storage performance of Ce(COOH)_3_-210 s compared with the other two materials. The specific capacities of all the three samples at each current density are calculated from the corresponding galvanostatic discharge curves, and the results are displayed in Figure 4d. The Ce(HCOO)_3_-150 s, Ce(HCOO)_3_-210 s, and Ce(HCOO)_3_-270 s electrodes demonstrate maximum specific capacities of 34.8, 132.0, and 39.7 F g^−1^ at 1 A g^−1^, respectively. It is noteworthy that the capacitance performance of the urchin-like Ce(HCOO)_3_-210 s holds a significant advantage compared to other reported Ce compounds, such as Ce(OH)_3_ thin films (75 F g^−1^ at 5 mV s^−1^) [50], CeO_2_ nanoparticles (89 F g^−1^ at 1 A g^−1^) [51], rod-like Ce-BTC (103 F g^−1^ at 1 A g^−1^) [52], and one-dimensional Ce(HPO_4_)_2_·xH_2_O (114 F g^−1^ at 0.2 A g^−1^) [33]. The highest specific capacitance of Ce(HCOO)_3_-210 s is ascribed to the optimal irradiation time, which leads to the highest crystallinity, SSA, and finest hierarchical structure with a mesoporosity that is beneficial for its conductivity, offering rich Ce^3+^/Ce^4+^ active sites and facilitating the transportation of electrolyte ions and thereby enhancing its charge-storage capability. In addition, the specific capacities of all the samples show a similar decreasing trend with the increase in the current density. This phenomenon is due to the electrolyte ions having sufficient time to contact and interact with the active material at a low current density, resulting in a high specific capacitance, whereas at a high current density, the intercalation/deintercalation process of electrolyte ions is too fast to limit the faradic redox reactions on the surface of the active material; thereby, the electrodes exhibit low specific capacitance values [53]. At a current density of up to 10 A g^−1^, the specific capacitance of the Ce(HCOO)_3_-210 s decreases to 82.3 F g^−1^. Compared to the value at 1 A g^−1^, the 62.3% of capacitance retention rate suggests its excellent rate capability.

The electrode/electrolyte interfacial charge transfer and capacitive properties of the Ce(HCOO)_3_-150 s, Ce(HCOO)_3_-210 s, and Ce(HCOO)_3_-270 s electrodes were studied by the electrochemical impedance spectroscopy (EIS). The EIS measurement was carried out in the frequency range of 1 Hz to 100 kHz, and the comparative Nyquist plots of all the three electrodes are displayed in Figure 4e. It is obvious that all the plots show similar profiles, with quasi-semicircular parts (the inset amplified diagram) in the high-frequency region and linear parts in the low-frequency region. The intercept of the Nyquist plot on the real (Z’) axis at the starting of the quasi-semicircle indicates the equivalent series resistance (R_s_), which mainly consists of the resistance of the electrode materials and the bulk KOH electrolyte. It can be seen from Figure 4e that all the three samples’ intercepts are very close to 0. The low R_s_ of the Ce(HCOO)_3_ electrodes is conducive to their rate capability and makes them more capacitive. Moreover, the diameter of the semicircle (or kinetic loop) in the Nyquist plot represents the charge transfer resistance (R_ct_), which originates from the discontinuity of the charge transfer process occurring at the electrode/electrolyte interface. As shown in the amplified diagram, the diameter of the Ce(HCOO)_3_-210 s electrode’s kinetic loop is smaller than that of the other two electrodes. The lowest R_ct_ of Ce(HCOO)_3_-210 s benefits from its fine hierarchical structure, which is composed of interconnected nanorods, and the rich mesoporosity that shortens the electron transfer path and facilitates the transportation of electrolyte ions. Actually, after being fitted with an equivalent circuit (the inset, where R_s_, R_ct_, CPE, and Z_w_ represent equivalent series resistance, charge transfer resistance, constant phase angle element, and Warburg impedance, respectively), using the ZView software, the R_ct_ values of the Ce(HCOO)_3_-150 s, Ce(HCOO)_3_-210 s, and Ce(HCOO)_3_-270 s electrodes are calculated to be 0.72, 0.63, and 0.66 Ω, respectively. These results are in good agreement with the previous structural analysis. The linear parts of the Nyquist plots in the low-frequency region signify the ideality of the samples towards pure capacitive behavior. The line closer to 90° along the imaginary (Z”) axis suggests more ideal capacitive behavior, while the resistive behavior is inferred when the line is closer to 0° along the real (Z’) axis [54]. Of all three samples, the inclinations of the linear parts are more than 45°, indicating good capacitive performance. The linear part of the Ce(HCOO)_3_-210 s presents an obviously lower inclination compared to the other two samples, which is due to the larger proportion of pseudocapacitive behavior arising from its high SSA, which causes more Ce^3+^/Ce^4+^ active sites to participate in redox reactions.

The electrochemical stability is the most promising indicator for evaluating the practical applicability of electrode materials. The Ce(HCOO)_3_-150 s, Ce(HCOO)_3_-210 s, and Ce(HCOO)_3_-270 s samples were subjected to 10,000 charge–discharge cycles at a constant current density of 10 A g^−1^. As shown in Figure 4f, the capacitances of all three electrodes increased in the initial 1000 cycles, then slowly decreased and eventually achieved outstanding capacity retention of over 100% even after 10,000 cycles. The initial increase in capacitance is believed to be related to the activation process of the electrode materials, and the ultra-high cyclic stability benefits from the intrinsic thermodynamic stability derived from their robust coordination bonds and kinetic stability, which is enabled by their unique urchin-like hierarchical structures. Additionally, it is worth noting that the capacitance of Ce(HCOO)_3_-210 s is much higher than that of the other two samples throughout their whole service life.

Furthermore, the EIS impedance analyses of the Ce(HCOO)_3_-150 s, Ce(HCOO)_3_-210 s, and Ce(HCOO)_3_-270 s electrodes were also performed after 10,000 charge–discharge cycles. According to the comparative Nyquist plots shown in Figure S3, the Ce(HCOO)_3_-210 s electrode still displays the smallest kinetic loop. The R_ct_ values of the Ce(HCOO)_3_-150 s, Ce(HCOO)_3_-210 s, and Ce(HCOO)_3_-270 s electrodes after 10,000 cycles were calculated to be 0.47, 0.19, and 0.21 Ω, suggesting that Ce(HCOO)_3_-210 s maintained the fastest charge transfer during the cyclic test. The OH^−^ ions transportation behaviors in the three types of electrodes were investigated before and after 10,000 charge–discharge cycles. Figure S4a,b present the linear relationship between Z’ and ω^−1/2^ (ω represents the angular frequency) in the low-frequency region of the Nyquist plots. The diffusion coefficients (D_OH−_) are calculated according to Equation (4) as follows:

(4) $$D_{OH^{-}}=\frac{R^{2}T^{2}}{2A^{2}n^{4}F^{4}C^{2}\sigma^{2}}$$

where R, T, A, n, F, C, and σ refer to the gas constant (J mol^−1^ K^−1^), absolute temperature (K), electrode surface area (cm^2^), number of transferred electrons, Faraday constant (96,485 C mol^−1^), ion concentration (mol L^−1^), and Warburg factor (Ω s^−1/2^), respectively [55]. The calculated D_OH−_ before 10,000 cycles of the Ce(HCOO)_3_-150 s, Ce(HCOO)_3_-210 s, and Ce(HCOO)_3_-270 s electrodes are 4.0 × 10^−18^, 2.0 × 10^−18^, and 3.0 × 10^−18^ cm^2^ s^−1^, respectively. These small differences originate from the slightly slower activation process of Ce(HCOO)_3_-210 s. However, after 10,000 cycles, the D_OH−_ of Ce(HCOO)_3_-210 s (5.9 × 10^−17^ cm^2^ s^−1^) is significantly higher than that of Ce(HCOO)_3_-150 s (3.0 × 10^−18^ cm^2^ s^−1^) and Ce(HCOO)_3_-270 s (1.7 × 10^−18^ cm^2^ s^−1^), revealing the Ce(HCOO)_3_-210 s electrode to have the fastest diffusion rate of OH^−^ electrolyte ions and the best kinetic behavior.

Therefore, it is evident from CV, CP, EIS, and cyclic tests that Ce(HCOO)_3_-210 s has more potential for supercapacitor electrodes among all three samples.

To better understand the charge storage mechanism and reaction kinetics of the urchin-like Ce(HCOO)_3_-210 s compound, the capacitive effect and diffusion-controlled contribution of the total energy storage were quantitatively analyzed using the CV curves, with scan rates from 1 to 30 mV s^−1^. According to Dunn’s approach [56], based on Equation (5) we calculate as follows:

(5) $$i_{v}=k_{1}v+k_{2}v^{1/2}$$

**Figure 5:**
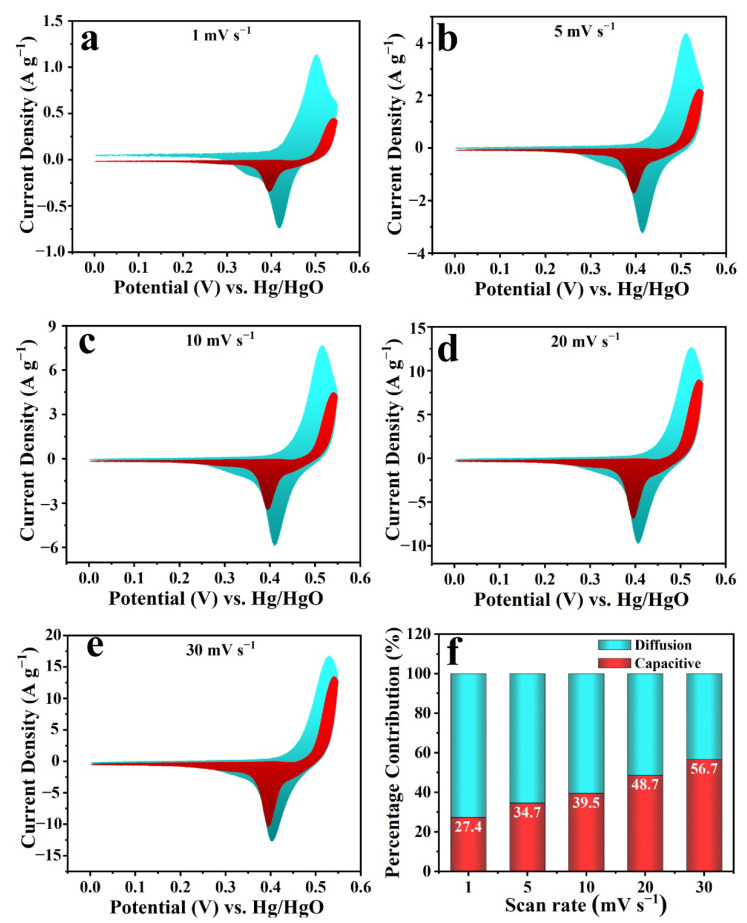
Analysis of charge storage mechanism and reaction kinetics of the urchin-like Ce(HCOO)_3_-210 s sample. (**a**) Scan rate: 1 mV s^−1^. (**b**) Scan rate: 5 mV s^−1^. (**c**) Scan rate: 10 mV s^−1^. (**d**) Scan rate: 20 mV s^−1^. (**e**) Scan rate: 30 mV s^−1^. (**f**) The relative contributions of specific capacitance from capacitive (blue) and diffusion-controlled (red) charge storage processes at various scan rates.

The current response at a certain potential (*i_v_*) originates from the capacitive effect with the fast kinetics (*k*_1_*v*) and diffusion-controlled reactions (*k*_2_*v*^1/2^). Fortunately, the values of *k*_1_ and *k*_2_ can be calculated by the slope of *i_v_*/*v*^1/2^ against *v*^1/2^, so that the capacitive contribution and diffusion-controlled contribution can be separated. Figure 5a–e show the typical cyclic voltammograms for the capacitive current (red) against the diffusion-controlled current (blue), and Figure 5f summarizes the normalized contribution ratios of the capacitive capacitance and diffusion-controlled capacitance at various scan rates. Under a scan rate of 1, 5, 10, 20, and 30 mV s^−1^, the capacitive portions are 27.4%, 34.7%, 39.5%, 48.7%, and 56.7%, respectively. Obviously, the capacitive contribution expands, and the diffusion-controlled contribution shrinks with the increase in scan rate. This can be ascribed to the fact that the capacitive capacitance derives from a pseudocapacitive faradaic reaction, which is a much faster electrochemical process than the electrostatic adsorption of electrolyte ions that enables the diffusion-controlled capacitance [54]. In addition, the diffusion of electrolyte ions may also be hindered by an insertion into the lattice of the Ce(HCOO)_3_-210 s crystals [57]. Therefore, the decrease in the diffusion-controlled capacitance becomes more pronounced at higher scan rates.

**Figure 6:**
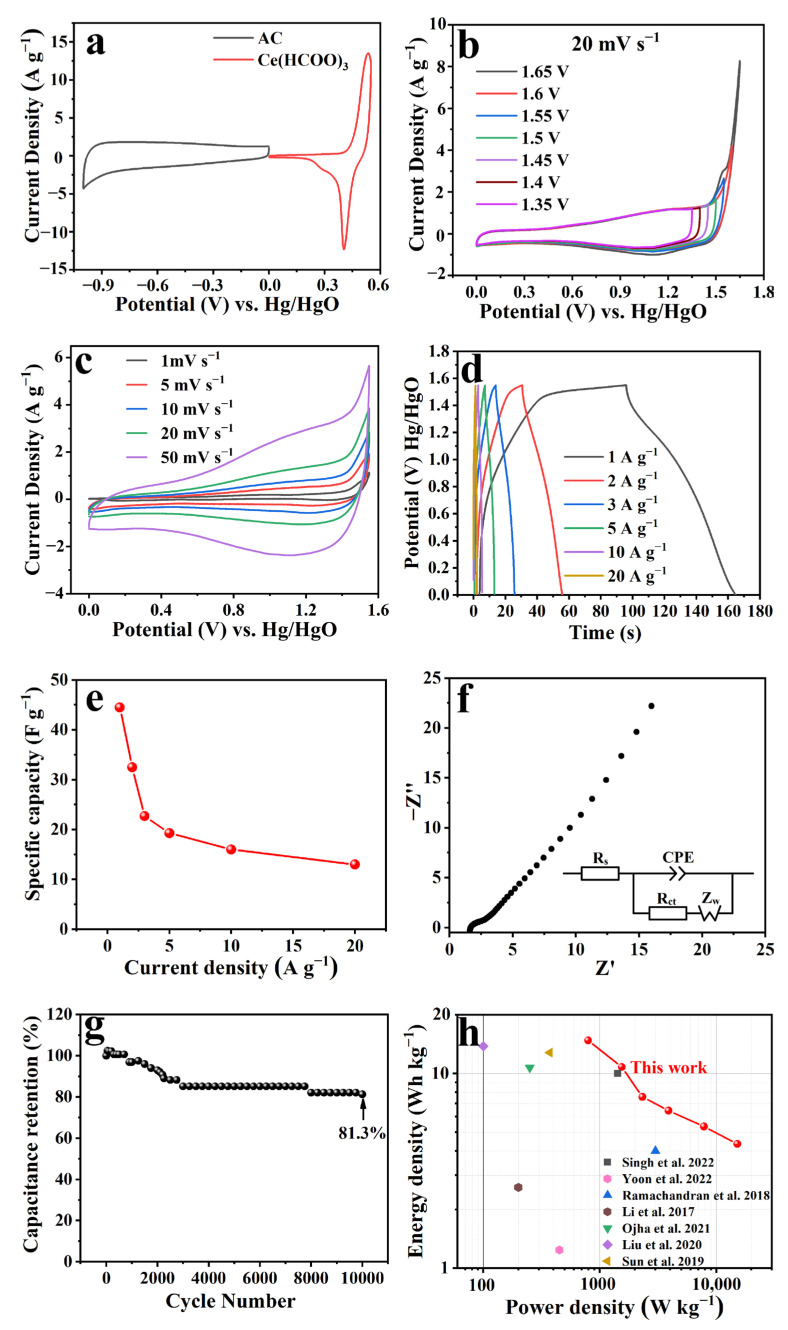
(**a**) Typical CV curves of the AC anode and Ce(HCOO)_3_ cathode in the potential range of −1 to 0.55 V. (**b**) CV profiles of the Ce(HCOO)_3_//AC-based ASC collected within different potential windows at scan rate of 20 mV s^−1^. (**c**) CV curves at various scan rates. (**d**) GCD profiles at different current densities. (**e**) Rate performance. (**f**) Nyquist plot. (**g**) Cyclic performance. (**h**) The comparison map of the power and energy densities of the ASC device [31,33,58,59,60,61,62].

Furthermore, it is well established that an ASC features both high power and energy densities, since it can fully utilize the opposite potential window that is derived from different positive and negative electrodes, making it highly promising for real-time applications. Along this line, we have fabricated a Ce(HCOO)_3_//AC-based ASC using a Ce(HCOO)_3_-210 s sheet as the positive electrode and an activated carbon sheet as the negative electrode. Its electrochemical properties were studied in a two-electrode test system. Figure 6a presents the CV curves of the AC negative electrode and the Ce(HCOO)_3_ positive electrode. It can be seen that the potential window of −1 to 0 V is contributed by AC and 0 to 0.55 V is due to Ce(HCOO)_3_. The CV curve of the AC negative electrode shows a quasi-rectangular shape, indicating a nearly ideal capacitive behavior. Meanwhile, the CV curve of the Ce(HCOO)_3_ positive electrode has a pair of distinct redox peaks, which is a feature of pseudocapacitive behavior. To verify the actual appropriate potential window of the Ce(HCOO)_3_//AC-based ASC, a series of CV curves at different voltage windows were investigated. As shown in Figure 6b, the CV profiles remain normal within 1.55 V. However, once the operating voltage exceeds 1.55 V, the CV curves exhibit an obvious polarization phenomenon due to the oxygen evolution reaction. Therefore, the optimal potential operating window of the ASC device can be extended to 1.55 V. Figure 6c demonstrates the CV curves of the ASC at various scan rates. The shape of the CV curve combines the characteristics of both electrodes and remains almost unchanged at all scan rates, suggesting the fast-charging transport behavior and excellent rate capability of the device. The GCD profiles for the ASC at different current densities are displayed in Figure 6d. The non-linear GCD curves reveal the pseudocapacitive characteristic of the Ce(HCOO)_3_ cathode, and the approximately symmetric shape signifies a favorable electrochemical reversibility of the ASC device. The specific capacitances of the ASC, calculated from the GCD profiles as a function of the current densities, are exhibited in Figure 6e. A high specific capacitance of 45 F g^−1^ based on the whole weight of the AC anode and the Ce(HCOO)_3_ cathode can be obtained at a current density of 1 A g^−1^. The specific capacitance of the ASC still remains 13 F g^−1^, even when the current density is magnified extremely by 20 times, indicating a good rate capability of the ASC device. Figure 6f shows the Nyquist plot and the corresponding equivalent fitting circuit of the ASC. The low intercept on the real (Z’) axis, small semicircle at high frequencies, and nearly vertical linear part at low frequencies suggest the low equivalent series resistance (R_s_ is 1.7 Ω), rapid ion transfer (R_ct_ is 1.1 Ω), and excellent capacitive behavior, respectively. The cycling stability of the ASC device was also evaluated by performing a long-term charging/discharging measurement at a high current density of 3 A g^−1^. As shown in Figure 6g, the specific capacitance of the ASC rapidly increases to 102.5% of its initial value in the first 200 cycles and then decreases gradually. After 10,000 cycles, a high capacitance retention rate of 81.3% can still be observed, revealing the outstanding electrochemical stability of the ASC device.

Finally, the energy and power density are dual essential aspects that determine the practical applications of supercapacitor devices. Therefore, the values of the energy and power density were also calculated, taking into account the total mass of the AC anode and the Ce(HCOO)_3_ cathode. Figure 6h displays the Ragone plot (energy density vs. power density plot) of the ASC device. It can be seen that the ASC can distribute a maximum energy density of 14.78 Wh kg^−1^ at a power density of 794.60 W kg^−1^ when the current density is 1 A g^−1^. As the current density increases to 20 A g^−1^, the energy density value gradually reduces and can still retain 4.34 Wh kg^−1^ at a considerably high power density of 15,168 W kg^−1^. It should be noted that the energy density, power density, and cyclic stability of the Ce(HCOO)_3_//AC-based ASC device are among the highest values for Ce–organic compound-based supercapacitors and also surpass or are comparable with many other reported electrode materials, as summarized in Table 3 [31,33,58,59,60,61,62]. To conclude, the excellent comprehensive energy storage properties of the ASC device are mainly ascribed to the high theoretical capacitance, optimum crystallinity, rapid redox capability, and most importantly, the unique urchin-like nanostructures of Ce(HCOO)_3_. The synergistic effect of these structural properties offers abundant Ce^3+^/Ce^4+^ active sites, enhanced electrolyte penetration, and adequate space to buffer the volume change during charging–discharging cycles, thereby significantly improving the electrochemical energy storage of this electrode material.

## 3. Materials and Methods

### 3.1. Materials

Ammonium cerium(IV) nitrate (Ce(NH_4_)_2_(NO_3_)_6_), N,N-Dimethylformamide (DMF), N-methyl-2-pyrrolidone (NMP), and formic acid (HCOOH) were purchased from Macklin chemical reagent Co., Ltd. (Shanghai, China). Ethylene glycol and ethyl alcohol were supplied by Xilong scientific Co., Ltd. (Shantou, China). Acetylene black and Polyvinylidene fluoride (PVDF) were procured from Tian jin Chemical Technology Co., Ltd. (Tianjin, China). Potassium hydroxide (KOH) was obtained from Sinopharm Chemical Reagent Co., Ltd. (Shanghai, China). All the analytical reagents were used without additional purification.

### 3.2. Fabrication of Ce(HCOO)_3_ Electrode and Ce(HCOO)_3_//AC-Based ASC

**Scheme 1:**
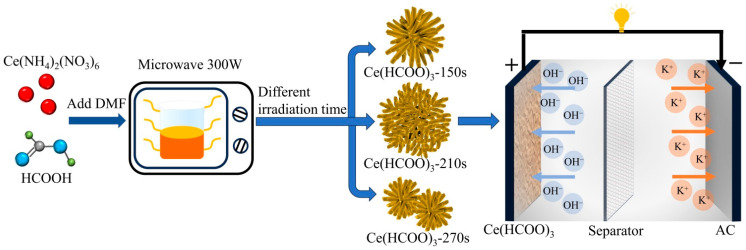
The synthetic route for Ce(HCOO)_3_-150 s, Ce(HCOO)_3_-210 s, Ce(HCOO)_3_-270 s, and Ce(HCOO)_3_//AC-based asymmetric supercapacitor.

The rapid microwave-assisted synthetic route for Ce(HCOO)_3_ samples and Ce(HCOO)_3_//AC-based asymmetric supercapacitor is shown in Scheme 1. In brief, 6.4 mM Ce(NH_4_)_2_(NO_3_)_6_ was solubilized in 12 mL ethylene glycol, and then 16 mL DMF was added and stirred for 30 min. After that, 41.2 mL HCOOH was added dropwise and stirred continuously to form a homogeneous solution. Then, this orange-colored mixture was transferred into a domestic microwave oven (Panasonic NN-GF352 M, 2450 MHz, Osaka, Japan) at a power of 300W, and the reaction was carried out for different periods, viz., 150, 210, and 270 s at each setting to optimize the process condition. When the reaction was complete and the mixture was cooled to room temperature, the products were separated by suction filtration and purified several times with ethanol and deionized water alternately. Subsequently, the samples were dried at 80 °C overnight and labeled as Ce(HCOO)_3_-150 s, Ce(HCOO)_3_-210 s, and Ce(HCOO)_3_-270 s, respectively.

The Ce(HCOO)_3_ electrodes were prepared by mixing 80% active material (Ce(HCOO)_3_-150 s, Ce(HCOO)_3_-210 s, and Ce(HCOO)_3_-270 s), 10% acetylene black, and 10% PVDF homogeneously using NMP as solvent. The obtained slurry was uniformly coated on a clean Ni foam (1 × 1 cm^2^) and dried at 80 °C overnight. The mass loading of Ce(HCOO)_3_ active material in each electrode was ~1 mg cm^−2^.

The asymmetric supercapacitor (ASC) device was fabricated using Ce(HCOO)_3_ active material as positive electrode, activated carbon (AC) as negative electrode, and 3M KOH as electrolyte. For the ASC assembly, the mass loading of the Ce(HCOO)_3_ in positive electrode (m^+^) and AC in negative electrode (m^−^) was determined according to the charge balance Equation (6) as follows:

(6) $$\frac{m^{+}}{m^{-}}=\frac{C_{sp}^{-}\times ∆V^{-}}{C_{sp}^{+}\times ∆V^{+}}$$

where $C_{sp}^{+}$ and $C_{sp}^{-}$ are the specific capacitance, and $∆V^{+}$ and $∆V^{-}$ are the operating potential window selected for positive and negative electrodes, respectively.

### 3.3. Structural Characterization, Morphological Study, and Electrochemical Measurement

The as-synthesized samples were characterized by X-ray diffraction (XRD, Rigaku Ultima IV, Rigaku Corporation, Tokyo, Japan) using Cu Kα radiation (*λ* = 1.5406 Å). Fourier transform infrared spectroscopy (FT-IR) was carried out in a Fourier transform infrared spectrometer (Thermo Fisher Nicolet 6700, Thermo Fisher, Waltham, MA, USA). Brunauer–Emmett–Teller (BET) analysis was performed on an Autosorb-iQ system (Quantachrome Instruments, Boynton Beach, FL, USA) at 77 K. The elemental compositions and energy states of the samples were examined by X-ray photoelectron spectroscopy (XPS, Escalab 250XI, Thermo Fisher Scientific, Marlborough, MA, USA).

The detailed morphological characteristics and elemental mapping of the samples were investigated by scanning electron microscopy (SEM, Hitachi SU-8100, Hitachi, Tokyo, Japan), with an energy-dispersive X-ray spectrometer attached (EDS, IXRF3310, IXRF Systems, Portland, OR, USA), and transmission electron microscopy (TEM, FEI Tecnai G2 F30, FEI Company, Hillsboro, OR, USA).

The electrochemical performance of the electrodes was evaluated using an electrochemical workstation (CHI 660E, Shanghai Chenhua Instrument Co., Ltd, Shanghai, China). Half-cell characteristics were investigated by a typical three-electrode system. The Ce(HCOO)_3_ active material was used as working electrode, Pt foil as counter electrode, Hg/HgO as reference electrode, and 3 M KOH as electrolyte, respectively. The specific capacitance of each sample was calculated from the corresponding GCD curve using Equation (7) as follows:

(7) $$C_{sp}=\frac{I\times \int Vdt}{m\times \int_{V_{i}}^{V_{f}}VdV}$$

where $C_{sp}$ represents the specific capacitance (F g^−1^), I indicates the discharge current (A), m refers to the mass of Ce(HCOO)_3_ active material (g), and *V*, *V_i_*, and *V_f_* denote the potential window (V), initial potential (V), and final potential (V), respectively.

The energy density and power density of each sample were calculated using Equations (8) and (9) as follows:

(8) $$E=\frac{I\times \int ∆V(t)\times ∆t}{m\times 3.6}$$

(9) $$P=\frac{E\times 3600}{∆t}$$

where E represents the energy density (Wh kg^−1^), P designates the power density (W kg^−1^), I is the constant current (A), ∫∆V(t) is the integral of GCD discharge area, ∆t is the discharge time (s), and m indicates the mass of Ce(HCOO)_3_ active material (g).

## 4. Conclusions

In this work, a facile microwave-assisted method was adopted to fabricate an urchin-like Ce(HCOO)_3_ structure composed of nanorods. The self-assembling of the urchin-like Ce(HCOO)_3_ clusters was tunable by controlling the irradiation time. The samples were characterized in terms of structural, morphological, and electrochemical properties, and the structure–property relationship was investigated. The successful synthesis of Ce(HCOO)_3_ rhombohedral crystals was confirmed by XRD, FT-IR, XPS, and HRTEM analyses. The crystallinity index, grain size, and SSA of the as-synthesized Ce(HCOO)_3_ were observed to increase from 150 to 210 s and then subsequently decrease from 210 to 270 s. The results of the morphology characterization matched well with the structural analysis. Ce(HCOO)_3_-210 s has the smallest sub-unit and finest 3D hierarchical structure. As expected, Ce(HCOO)_3_-210 s exhibits the best comprehensive electrochemical performance among the tested samples, which can be attributed to its optimal crystallinity and mesoporous hierarchical structure that are beneficial for its conductivity, offering numerous Ce^3+^/Ce^4+^ active sites and facilitating the electrolyte penetration. The maximum specific capacitance of 132 F g^−1^ was achieved for this sample at the current density of 1 A g^−1^. Moreover, an asymmetric supercapacitor based on Ce(HCOO)_3_//AC was fabricated, and it delivers a maximum energy density of 14.78 Wh kg^−1^ and a maximum specific power density of 15,168 W kg^−1^. The ASC device also shows outstanding electrochemical stability by achieving a high capacitance retention rate of 81.3% after 10,000 charge–discharge cycles at 3 A g^−1^. These excellent electrochemical properties demonstrate that the urchin-like Ce(HCOO)_3_ is a promising electrode material for supercapacitors.

## Figures and Tables

**Table 1:** The crystallinity index and average grain size of the Ce(HCOO)_3_ samples as a function of irradiation time.

Sample	Crystallinity Index	Average Crystallite Size (nm)
Ce(HCOO)_3_-150 s	39.24%	34.27
Ce(HCOO)_3_-210 s	86.67%	44.19
Ce(HCOO)_3_-270 s	65.93%	40.85

**Table 2:** The specific surface area, BJH pore volume, and average pore size of the Ce(HCOO)_3_ samples synthesized under different irradiation time.

Sample	Specific Surface Area (m^2^ g^−1^)	BJH Pore Volume (cm^3^ g^−1^)	Average Pore Size (nm)
Ce(HCOO)_3_-150 s	1.93	0.015	3.796
Ce(HCOO)_3_-210 s	2.89	0.023	3.391
Ce(HCOO)_3_-270 s	2.57	0.020	3.803

**Table 3:** Comparison of the electrochemical performances of Ce(HCOO)_3_//AC-based ASC and other recently reported metal–organic compound-based supercapacitors.

Materials	Energy Density (Wh kg^−1^)	Power Density (W kg^−1^)	Cyclic Stability (Cycle Numbers)	Ref.
Ce-H_2_L//Ce-H_2_L	10	1425	70% (5000)	[31]
Ce(HPO_4_)_2_·xH_2_O//Ce(HPO_4_)_2_·xH_2_O	1.24	449.8	92.7% (5000)	[33]
Ce-BTC//Ce-BTC	4	3000	83% (5000)	[58]
Cu–CAT NWAs//Cu–CAT NMAs	2.6	200	85% (5000)	[59]
Cr-MOF//BPC	10.7	250	85% (10,000)	[60]
Cu-BDC//Cu-BDC	13.8	100	80% (2000)	[61]
Ni/Co-MOF//AC	12.8	372.5	70.3% (2000)	[62]
Ce(HCOO)_3_//AC	14.78	794.6	81.3% (10,000)	This work

## Data Availability

The data presented in this study are available on request from the corresponding author. The data are not publicly available due to commercial reasons.

## Supplementary Materials

The following supporting information can be downloaded at https://www.mdpi.com/article/10.3390/molecules29020420/s1: Figure S1. The definition of *a*, *b*, *c* and *l* for calculation of crystallinity index (CI) from XRD spectra. Figure S2. GCD curves of the (a) Ce(HCOO)_3_-150s, (b) Ce(HCOO)_3_-210s and (c) Ce(HCOO)_3_-270s at various current densities. Figure S3. Comparative Nyquist impedance spectra of the Ce(HCOO)_3_-150 s, Ce(HCOO)_3_-210 s and Ce(HCOO)_3_-270 s samples after 10,000 charge-discharge cycles, and the inset is the magnified part for the high-frequency region as well as the equivalent circuit diagram. Figure S4. Linear relationship between Z′ and ω^−1/2^ in the low frequency region (a) before 10,000 charge-discharge cycles and (b) after 10,000 charge-discharge cycles.
