# Supplementary material for: Urchin-like Ce(HCOO)3 Synthesized by a Microwave-Assisted Method and Its Application in an Asymmetric Supercapacitor
Source: Molecules. 2024 Jan 15;29(2):420. doi: 10.3390/molecules29020420 (PMC10820376; doi:10.3390/molecules29020420)
Supplement: Supplementary file 1 [file molecules-29-00420-s001.zip › molecules-2801700-supplementary.pdf]

Supplementary Information

Urchin-like $Ce(HCOO)_3$ synthesized by microwave-assisted method and its application in asymmetric supercapacitor

Qing He [1,*], Wanglong Wang [2], Guohua Li [3], Wenmiao Chen [2], Xing Yang [1], Chengyuan Ni [1], Xing Fang [1,*]

[1] Key Laboratory of Air-driven Equipment Technology of Zhejiang Province, Quzhou University, Quzhou 324000, China; helinqi@163.com (Q.H.); yang7092481@163.com (X.Y.); nichengyuan1@126.com (C.N.); 36092@qzc.edu.cn (X.F.)

[2] Department of Mechanical Engineering, Zhejiang University of Technology, Hangzhou 310058, China; 221122020226@zjut.edu.cn (W.W.); 15714178548@163.com (W.C.)

[3] R&D Department, Quzhou Hixee Electronic Technology Co., Ltd., Quzhou 324000, China; bullcar@163.com (G.L.)

* Correspondence: helinqi@163.com (Q.H.); 36092@qzc.edu.cn (X.F.)

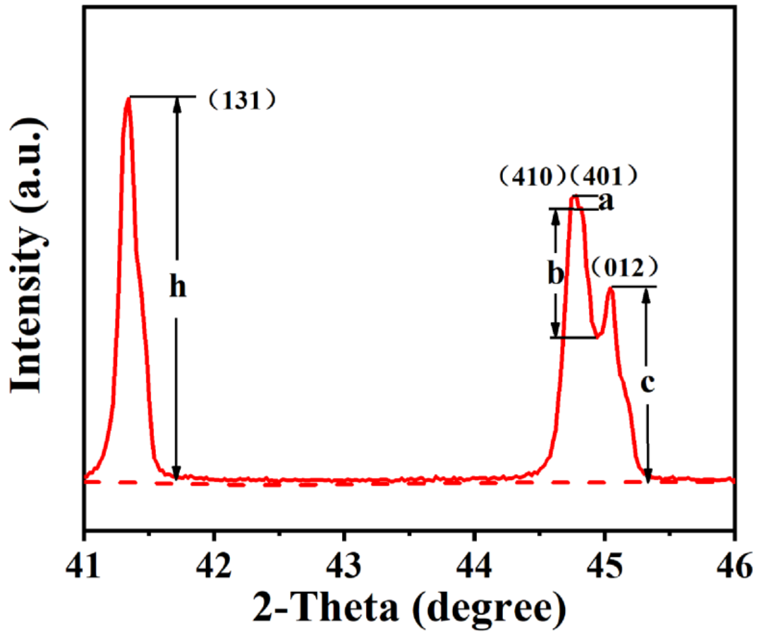

Figure S1. The definition of *a*, *b*, *c* and *l* for calculation of crystallinity index (CI) from XRD spectra.

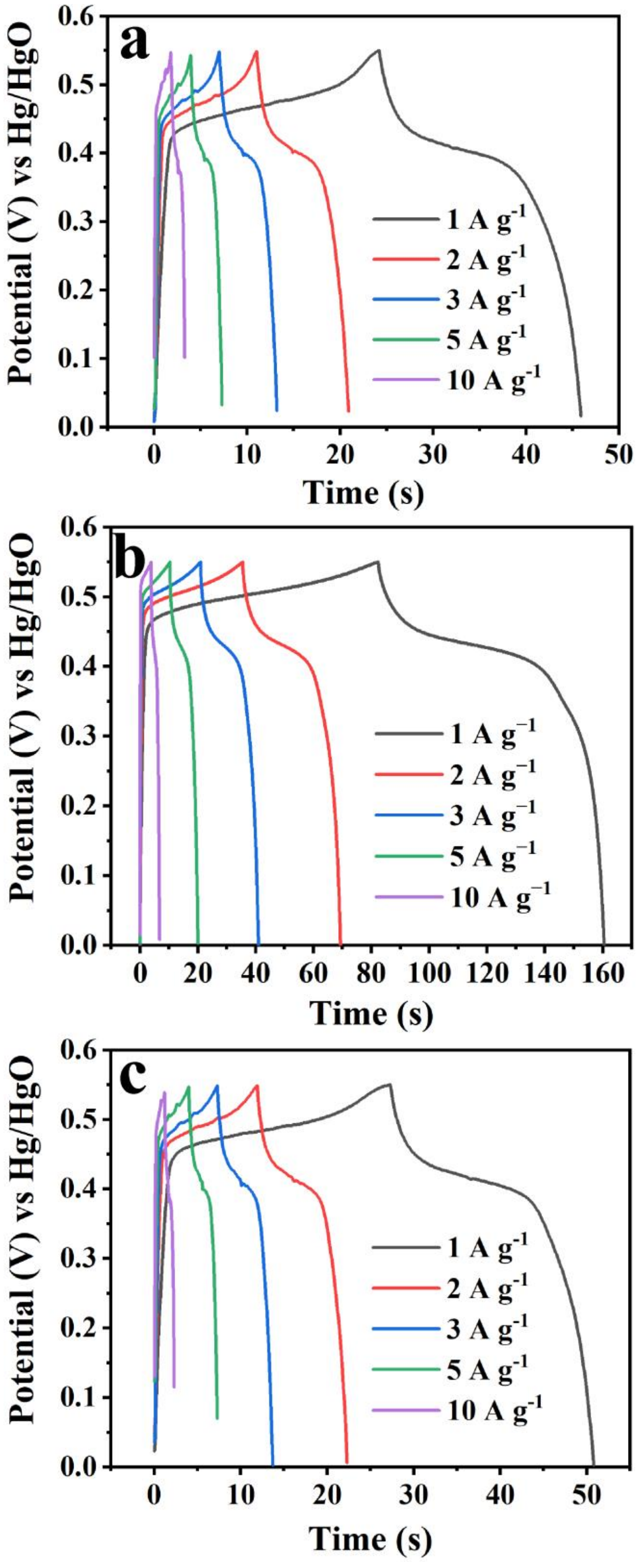

Figure S2. GCD curves of the **(a)** $Ce(HCOO)_3$-150s, **(b)** $Ce(HCOO)_3$-210s and **(c)** $Ce(HCOO)_3$-270s at various current densities.

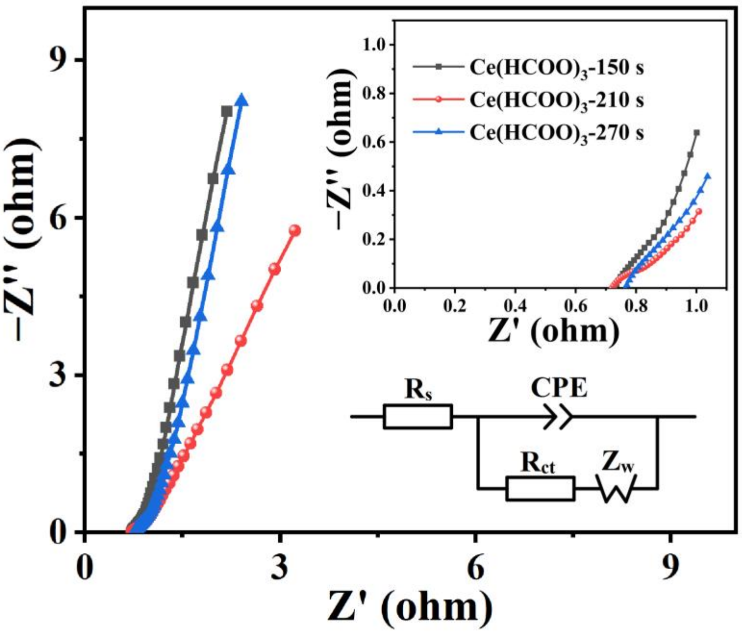

Figure S3. Comparative Nyquist impedance spectra of the $Ce(HCOO)_3$-150s, $Ce(HCOO)_3$-210s and $Ce(HCOO)_3$-270s samples after 10,000 charge-discharge cycles, and the inset is the magnified part for the high-frequency region as well as the equivalent circuit diagram.

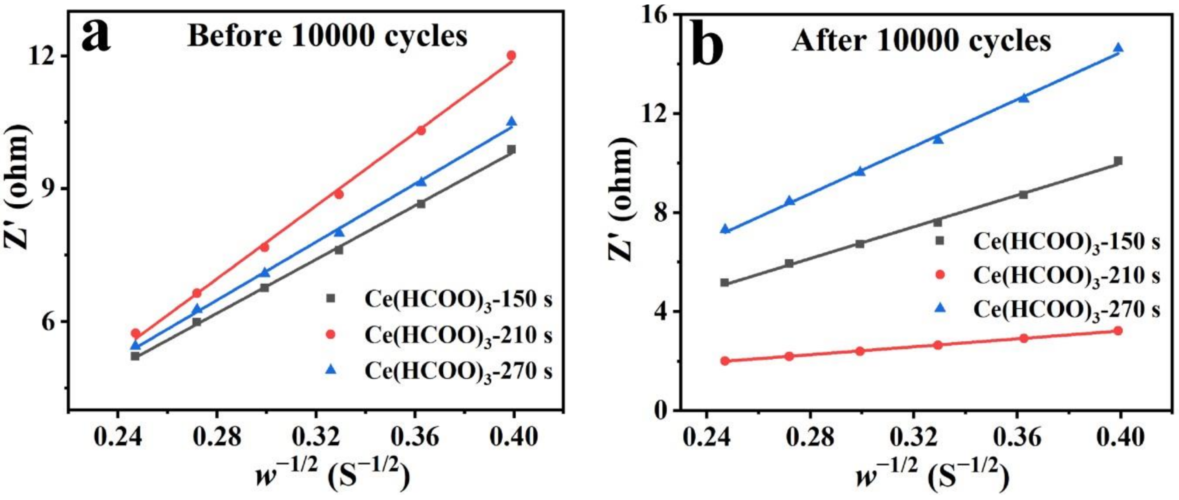

Figure S4. Linear relationship between Z' and $\omega^{-1/2}$ in the low frequency region **(a)** before 10,000 charge-discharge cycles and **(b)** after 10,000 charge-discharge cycles.
